# Sodium butyrate regulates macrophage polarization by TGR5/β-arrestin2 in vitro

**DOI:** 10.1186/s10020-025-01096-7

**Published:** 2025-01-29

**Authors:** Miao Liu, Wen-jie Xie, Xu Zhang, Wei Wu, Guang Li, Lu Wang

**Affiliations:** 1 Department of Gastroenterology, Renmin Hospital of Wuhan University, Wuhan, 430060 Hubei China; 2 Department of Critical Care Medicine, Renmin Hospital of Wuhan University, 238 Jiefang Road, Wuchang, Wuhan, 430060 Hubei China; 3 Central laboratory, Renmin Hospital of Wuhan University, Wuhan, 430060 Hubei China

**Keywords:** Sodium butyrate, Macrophage polarization, TGR5, β-arrestin2

## Abstract

**Background:**

Macrophages play an important role in the pathogenesis of ulcerative colitis (UC). We will explore the effects of sodium butyrate (SB) on macrophage function.

**Methods:**

The targets of butyric acid were identified using SwissTargetPrediction database and surface plasmon resonance (SPR). Limited proteolysis mass spectrometry (Lip-MS) was used to further investigate the binding sites of butyric acid with its targets and molecular docking was employed to simulate their binding modes. Macrophage polarization model was established with lipopolysaccharide (LPS) in vitro. Takeda G protein-coupled receptor 5 (TGR5) and β-arrestin2 expression and macrophage polarization markers were detected with or without SB.

**Results:**

TGR5 was identified as the target of butyric acid. Moreover, the amino acid regions 275–286 and 321–330 of TGR5 (GPBAR1 [275–286] and GPBAR1 [321–330]) were the potential binding regions for butyric acid. Based on molecular docking analysis, butyric acid formed effective hydrogen-bonding interactions with ASP-284 and TYR-287 of TGR5. In cell experiments, LPS inhibited the expression of TGR5, β-arrestin2, IL-10, ARG1, and CD206 and increased the expression of IL-1β, iNOS, and CD86, while SB reversed the effect of LPS. SBI-115, a TGR5 antagonist, and knockdown of β-arrestin2 inhibited the effect of sodium butyrate. INT-777, a TGR5 agonist, reversed the inhibitory effect of knockdown of β-arrestin2.

**Conclusion:**

SB inhibited M1-like polarization and promoted M2-like polarization induced by LPS via TGR5/β-arrestin2 in RAW264.7 cells and TGR5 was the target of SB.

## Background

UC is a chronic inflammatory condition of the colon that reduces the quality of life for patients. About 1.5 million people in North America have been diagnosed with ulcerative colitis, making it a significant contributor to public health concerns (Voelker 2024). UC most commonly occurs in individuals between the ages of 20 to 40 years. Moreover, patients diagnosed with UC have a 4.5% risk of developing colon cancer within 20 years, which is 1.7 times higher than that of the general population (Voelker 2024). Delving into its pathogenesis and treatment strategies can help better control disease prevalence, alleviate patient suffering, and reduce socioeconomic burdens. Our previous studies have confirmed that Clostridium butyricum has a protective effect on DSS-induced colitis and colitis-associated colon cancer in vivo (Liu et al. 2020a, b). However, the protective mechanism of Clostridium butyricum on UC needs further investigation. Intestinal immune cells can prevent the entry of pathogens or the translocation of intestinal microorganisms and maintain immune tolerance to the intestinal microbiota, which are associated with the cellular and molecular mechanisms of UC (Saez et al. 2023). Macrophages are an important component of intestinal immune cells and play a crucial role on the pathophysiology of UC (Saez et al. 2023). The main metabolites of Clostridium butyricum include organic acids such as butyric acid, lactic acid, and acetic acid, which play an important role in maintaining intestinal health. Whether these metabolites exert effects on macrophages and how their mechanisms of action work require further research.

TGR5 is also known as G protein-coupled bile acid receptor 1 (GPBAR1), which can be detected in human gastrointestinal tract, gallbladder, and immune tissues (Kawamata et al. 2003). TGR5 plays an important role in regulating bile acid metabolism, glucose and energy metabolism, inflammation and tumor (Sato et al. 2007). The study has found that TGR5 activation can promote the expression of tight junction protein and alleviate inflammatory bowel disease (Zhai et al. 2021), furthermore, can reduce inflammatory factors and inhibit TNF-α in mouse and human intestinal macrophages, leading to the innate immunosuppression of inflammatory bowel disease (Yoneno et al. 2013). β-arrestin2 (Arrb2) is a member of the arrestin family and mainly regulates the desensitization, internalization, and transport of G-protein coupled receptors (GPCRs). Arrestins can also be used as a scaffolding protein to recruit multiple non-G protein effectors and activate downstream signaling pathways (Ma et al. 2021). β-arrestin2 can attenuate colitis induced by dextran sulfate sodium (DSS) through modulation of T-cell activation (Sharma et al. 2015). Oxytocin binding to oxytocin receptor regulates macrophage polarization and reduces intestinal inflammation through up-regulation of β-arrestin2 (Tang et al. 2019). Gentiopicroside can inhibit renal inflammation by regulating TGR5/β-arrestin2 in diabetes mice (Xiao et al. 2020). Whether TGR5/β-arrestin2 pathway can regulate macrophage polarization to inhibit intestinal inflammation still needs further research.

SB, a short-chain fatty acid, is primarily produced by bowel microbial fermentation of dietary fibers (Chen et al. 2018), which serves as the energy source for intestinal epithelial cells, inhibition of epithelial cell inflammation, intestinal immune regulation and so on (Hamer et al. 2008). Studies have shown that butyrate mainly acts as a histone deacetylase inhibitor (HDACI) to regulate oxidative stress (Shimazu et al. 2013) and acts as a ligand for GPCRs to activate downstream pathways (Thangaraju et al. 2009). In this study, we will explore that whether SB can regulate the macrophage polarization by TGR5/β- arrestin2 in vitro.

## Materials and methods

### Target prediction and validation

TGR5 (Human GPBAR1-(AA 1-330)-Q8TDU6-Full length, MW 36800, cat. no. CSB-CF819471HU) was purchased from Yangene Biotechnology Co., Ltd. We searched for SMILES of butyric acid (MW 88.11, cat. no. B103500, Sigma-Aldrich, Merck KGaA) through PubChem and obtained potential targets for butyric acid by SwissTargetPrediction database inputting SMILES of butyric acid and selecting Homo sapiens. We validated the target of butyric acid through surface plasmon resonance (SPR, Biacore T200, GE) assay, as follows: The activator was prepared by mixing 400 mM 1-Ethyl-3-(3-dimethylaminopropyl) carbodiimide hydrochloride (EDC, cat. no. E1769-25G, Sigma-Aldrich, Merck KGaA) and 100 mM N-Hydroxysuccinimide (NHS, cat. no. 130672-25G, Sigma-Aldrich, Merck KGaA) immediately prior to injection. The CM5 sensor chip (cat. no. 29149603, GE) was activated for 600 s with the mixture at a flow rate of 10 µL/min. We used immobilization buffer (10 mM Sodium Acetate, pH 5.0) to dilute the target protein to 50 µg/mL and injected it to sample channel (Fc4) at a flow rate of 10 µL/min to reach immobilization levels of 2400 resonance units (RU). The chip was deactivated by 1 M Ethanolamine hydrochloride (cat. no. E6133-100G, Sigma-Aldrich, Merck KGaA) at a flow rate of 10 µL/min for 420 s. We utilized the analyte buffer [HEPES (pH 7.4): 10 mM HEPES, 150 mM NaCl, 3 mM EDTA, 0.05% P20] to dilute butyric acid (cat. no. B103500, Sigma-Aldrich, Merck KGaA) to different concentrations (125, 62.5, 31.25, 15.625, 7.8, and 0 µM). Butyric acid was injected to channel Fc1- Fc4 at a flow rate of 30 µL/min for an association phase of 90 s, followed by 180 s dissociation. The association and dissociation process were carried out in the analyte buffer. We repeat 6 cycles in the order of increasing analyte concentration.

### Limited proteolysis-mass spectrometry

Sample preparation: The experiment is divided into two groups: butyric acid group and control group. We mixed butyric acid (100 µM) or equal volume solvent with TGR5 (10 µg) and incubated them for 15 min at 25℃. Protease K was added to protein solution at a proteinase K to protein mass ratio of 1:100. We incubated the mixture for 3 min at 25℃, and immediately heat it at 98℃ for 5 min to terminate the digestion reactions. The samples after enzymatic hydrolysis were cooled to room temperature, and then equal volume of 2% sodium deoxycholate (SDC, dissolved by 20 mM Tris HCl) was added. The pH was adjusted to 7-8.5 with ammonium bicarbonate. The samples were heated at 98℃ for 5 min, cooled to room temperature and then reacted with 5 µL TCEP (0.1 M) and 5 µL chloroacetamide (0.4 M) in a dark environment at 45℃ and 1500 rpm for 5 min. The samples were taken out and cooled to room temperature, then were digested by trypsin (Promega, Madison, WI) at an enzyme substrate ratio of 1:50 overnight at 37℃. The samples were added an appropriate amount of formic acid (final concentration of 1.5%), mixed well and centrifuged at 16,000 g for 5 min to obtain the supernatant. Then the mixtures were desalted by C18 desalination column, vacuum dried, solubilized in 0.1% formic acid, and immediately analyzed by mass spectrometry using Exploris 480 mass spectrometer (Thermo Fisher Scientific, MA, USA). Tandem mass spectrometry was analyzed by Proteome Discoverer (Thermo Fisher Scientific, MA, USA).

### Molecular docking

The AlphaFold Protein Structure Database was used to obtain the three-dimensional structure of TGR5 through the input of UniProt accession (Q8TDU6). Molecular docking was completed by Covalent Docking in the Glide module of Schrödinger Maestro software. The Protein Preparation Wizard module was used to prepare, optimize, and minimize the receptor through constrained minimization using the OPLS3e force field. All molecules were prepared according to the default settings of the LigPrep module. When performing screening in the Glide module, the prepared receptor was imported to specify the appropriate position for receptor grid generation. The predicted site of the protein was selected as the centroid of a 12 Å box. Finally, molecular docking and screening were performed through standard docking methods.

### Molecular dynamics (MD) simulation

Gromacs 2020 software package was employed to perform molecular dynamics simulation for the screened receptor-ligand complex. The protein employed the AMBER99SB-ILDN force field parameters, while the small-molecule ligand adopted the GAFF force field parameters, and the topology of the small molecule was constructed using the Sobtop program, along with charge fitting conducted by RESP. The TIP3P water model was selected, with the minimum distance between the protein and the edge of the water box being 1.0 nm. The system charge was neutralized using sodium ions or chloride ions according to the docking results. The molecular dynamics simulation workflow consists of four steps, namely, energy minimization, heating, equilibration, and production dynamics simulation. The calculation of binding free energy between the ligand and the protein employed the gmx_MMPBSA method within the Gromacs 2020 program.

### Cells and preparation of medium

The RAW264.7 cells were purchased from Procell Life Science&Technology Co., Ltd (Wuhan, Hubei province, China) and were cultured in DMEM medium (Gibco) supplemented with 10% fetal bovine serum (FBS, Gibco), 1% Penicillin-Streptomycin Solution at 37 °C in a humidified 5% CO_2_ atmosphere. LPS (100 ng/mL, Sigma-Aldrich; Merck KGaA) was used to induced siRNA-β-arrestin2-transfected and non-transfected RAW264.7 cell polarization for 24 h. SB (50 µg/mL) was administered to the RAW264.7 cells followed by 2 h after LPS administration. INT-777 (cat. no. HY-15677, MCE, USA), a TGR5 agonist, was mixed with DMSO and diluted to a final concentration of 3 µM, followed by 2 h after LPS administration. SBI-115 (cat. no. HY-111534, MCE, USA), a TGR5 antagonist, was mixed with DMSO and diluted to a final concentration of 100 µM, followed by 2 h after LPS administration.

### Cells transfection with siRNA

siRNA-β-arrestin2 was transfected into RAW264.7 cells to knock down β-arrestin2 in RAW264.7 cells. siRNA-β-arrestin2 and negative siRNA-control (siRNA-NC) were synthesized by Jintuosi Biotechnology Co., Ltd in Wuhan, China. Three siRNA sequences were designed for the β-arrestin2 target gene as follows: siRNA-Arrb2(M)-568: sense: 5’-GGCUUGUGGAGUAGACUUUTT-3’ antisense: 5’-AAAGUCUACUCCACAAGCCTT-3’; siRNA-Arrb2(M)-916: sense: 5’-GGCUCAGCUAGAACAAGAUTT-3’ antisense: 5’-AUCUUGUUCUAGCUGAGCCTT-3’; siRNA-Arrb2(M)-1024: sense: 5’-GGAUGGGCAGCUCAAACAUTT-3’ antisense: 5’-AUGUUUGAGCUGCCCAUCCTT-3’. RAW264.7 cells were incubated in 6 well plates at 37 °C in 5% CO_2_ atmosphere overnight and cultured in serum-free DMEM medium 2 h before transfection. We used 100 µL serum-free opti-MEM (31985-070, Gibco) to dilute 10 µL siRNA (20 µM) and 5 µL LipofectamineTM 2000 (11668-019, Invitrogen) respectively and allowed the solution to stand at room temperature for 5 min. We mixed a diluent of LipofectamineTM 2000 and siRNA and allowed the solution to stand at room temperature for 20 min. RAW264.7 cells were incubated with siRNA diluent at 37 °C in 5% CO_2_ atmosphere for 6 h and then cultured in DMEM medium with FBS. After 24 h of transfection, the siRNA knockdown efficiency was identified using qRT-PCR.

### Quantitative real-time PCR (qRT-PCR)

We used qRT-PCR to detect mRNA expression of macrophage polarization markers. We used Trizol reagent (cat. no. 15596026; Invitrogen; Thermo Fisher Scientific, Inc.) to extract total RNA from RAW264.7 cells. We utilized HiScript II Q Select RT SuperMix for qPCR kit (cat. no. R233; Vazyme Biotech Co., Ltd.) to get rid of DNA for 2 min at 42 ˚C and convert RNA to cDNA according to following conditions: 50˚C for 15 min, 85˚C for 5 s, 4˚C for 10 min. Then we used AceQ qPCR SYBR Green Master Mix kit (cat. no. Q111‑02; Vazyme Biotech Co., Ltd.) for qRT-PCR detection on a QuantStudio 6 Flex Real‑Time PCR system (Applied Biosystems; Thermo Fisher Scientific, Inc.) according to following conditions: 95˚C for 10 min, 40 cycles of 95˚C for 15 s and 60˚C for 30 s, 95˚C for 15 s and 60˚C for 60 s and 95˚C for 15 s. We adopt 2-ΔΔ Ct method for quantitative analysis of the data. The primer sequences for target genes were displayed in Table 1.

**Table 1:** Primer sequences

Gene	Primer	Sequence (5’-3’)	PCR Products
GAPDH	Forward	ATGGGTGTGAACCACGAGA	229 bp
	Reverse	CAGGGATGATGTTCTGGGCA	
CD206	Forward	AACAAAGGGACGTTTCGGTG	180 bp
	Reverse	TCCTTCTGCCCAATGTTTGC	
CD86	Forward	GCCTCTCTCTTTCATTCCC	268 bp
	Reverse	CTGTCAGCGTTACTATCCCG	
IL-1b	Forward	AGGCAGTATCACTCATTGTGG	223 bp
	Reverse	ACGAGGCTTTTTTGTTGTTC	
IL-10	Forward	GCTGGACAACATACTGCTAACCG	218 bp
	Reverse	CACAGGGGAGAAATCGATGACAG	
iNOS	Forward	TTGGCTCCAGCATGTACCCT	121 bp
	Reverse	TCCTGCCCACTGAGTTCGTC	
ARG1	Forward	ATCGTGTACATTGGCTTGCG	184 bp
	Reverse	CGTCGACATCAAAGCTCAGG	

### Immunofluorescence staining

We rinsed the round coverslips with cultured cells using phosphate buffered saline (PBS) in the plate. The cultured cells were placed in 4% paraformaldehyde (cat.no.80096618, Sinopharm Chemical Reagent Co., Ltd.) for 15 min and rinsed for 3 times with PBS. Next, the fixed cells were permeabilized with 0.5% Triton X-100 (cat.no.ST795, Beyotime, China) for 20 min at room temperature, and rinsed for 3 times with PBS. We added 10% normal Goat Serum (cat.no.AR1009, Wuhan Boster Biological Technology co., Ltd) on the round coverslips for 30 min at room temperature. Then, cells were added enough diluted primary antibody: CD86 (cat.no.13395-1-AP, Wuhan Proteintech Group, Inc.), CD206 (cat.no.18704-1-AP, Wuhan Proteintech Group, Inc.) at 4 °C overnight, rinsed with PBS, and incubated with CY3 Conjugated AffiniPure Goat Anti-Rabbit IgG (H + L) (BA1032, Wuhan Boster Biological Technology co., Ltd) for 1 h at 37 °C. Cells were incubated with DAPI (cat.no.C1002, Beyotime, China) for 5 min in dark. The images were captured with a fluorescence microscope (Olympus, Yokohama, Japan).

### Western blot analysis

After RAW264.7 cells were administered with LPS with or without INT-777 and SBI-115 for 24 h, we utilized phenylmethanesulfonyl fluoride (PMSF, P105539, Shanghai Aladdin Bio‑Chem Technology Co., Ltd) to lyse cells and extract the total protein. BCA kit (P0010, Beyotime, China) was used to detect the protein concentration. The protein was separated by electrophoresis through 12% SDS-PAGE gel and transferred to polyvinylidene difluoride (PVDF) membranes. Then PVDF membranes were incubated with Tris buffered saline containing 0.1% Tween‑20 (TBST) including 5% skimmed milk for 2 h at room temperature. Primary antibodies were used to incubate the membranes overnight at 4 °C. Primary antibodies were as follows: GAPDH (37 kDa, AB-P-R 001, Hangzhou Goodhere Biotechnology Co., Ltd.), TGR5 (33 kDa, ab72608, Abcam), β-arrestin2 (50 kDa, ab54790, Abcam). The PVDF membranes were washed by TBST and further incubated with HRP-conjugated AffiniPure Goat anti-mouse secondary antibody (BA1051; Wuhan Boster Biological Technology, Ltd.) and HRP conjugated AffiniPure goat antirabbit secondary antibody (BA1054; Wuhan Boster Biological Technology, Ltd.) for 2 h at room temperature. Finally, ECL kit (P1050; Beijing Applygen Technologies Inc.) was used to visualize the protein bands. We made use of BandScan v5.0 software (Glyko Biomedical Ltd.) to analyse gray-scale values of the blots.

### Statistical analysis

All data were presented as mean ± standard deviation. Student’s t test was used to analyse the differences between two groups. Differences among multiple groups were analysed by one‑way ANOVA, followed by Tukey’s post‑hoc test. *P*-value < 0.05 was statistically significant. Part figures were made in STRING (version 12.0) (string-db.org).

## Results

### The target of butyric acid was TGR5

**Fig. 1:**
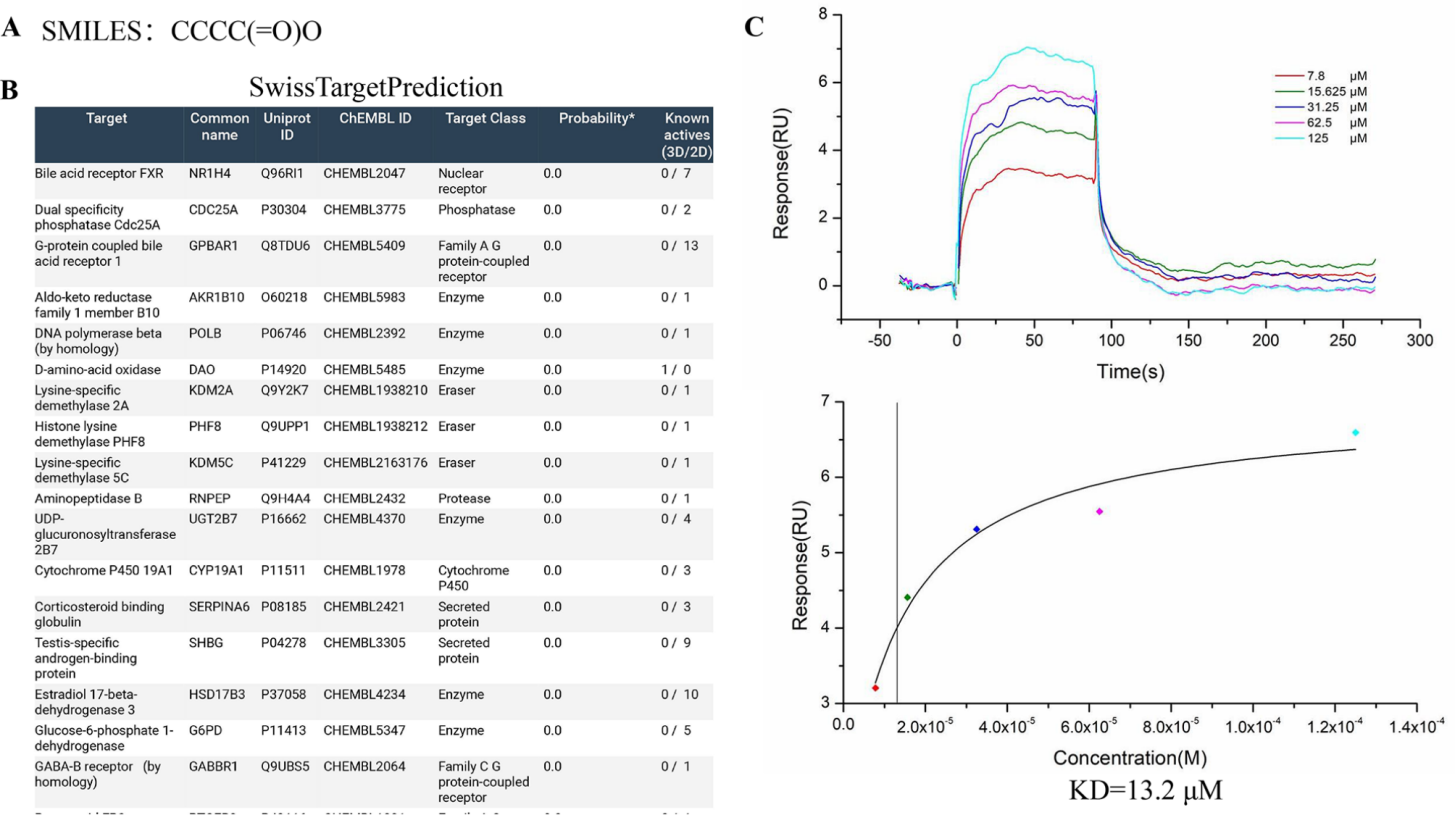
Prediction and verification of the target of butyric acid. (**A**) The SMILES of butyric acid was retrieved in Pubchem. (**B**) GPBAR1 was predicted as a target of butyric acid by SwissTargetPrediction. (**C**) SPR was used to verify the interaction between butyric acid and GPBAR1 (TGR5). GPBAR1, G protein-coupled bile acid receptor 1; TGR5, Takeda G protein-coupled receptor 5; SPR, surface plasmon resonance

To find the target of butyric acid, we found that the SMILES of butyric acid in PubChem system (Fig. 1A) was CCCC(= O)O. We used the SwissTargetPrediction database (Fig. 1B) to obtain the target prediction result, which showed that a potential target was GPBAR1 (TGR5) associated with digestive system. The affinity constants of GPBAR1 and butyric acid were determined by SPR assay (Fig. 1C), and we successfully elucidated that GPBAR1 captured on the CM5 chip could bind to butyrate, with an affinity constant determined in the SPR assay to be 13.2 µM.

### Identification of the binding site between butyric acid and TGR5 using LiP-MS

**Fig. 2:**
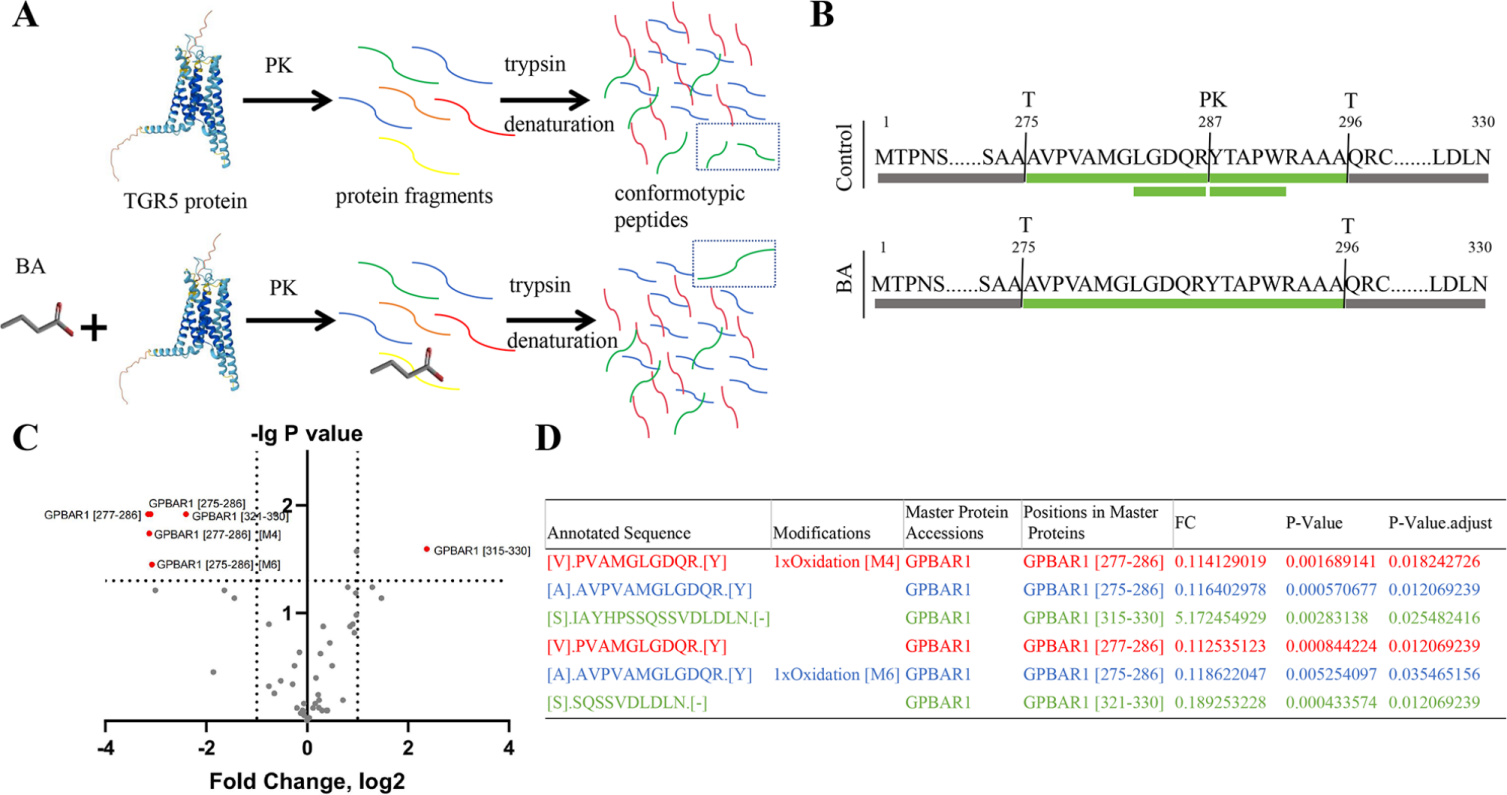
The binding site of butyric acid and TGR5 revealed by LiP-MS. (**A-B**) the workflow of the LiP-MS: (**A**) TGR5 was treated with or without butyric acid and might undergo local conformational changes induced by butyric acid. Under natural conditions, limited proteolysis was carried out with PK, and the protein fragments were completely digested by trypsin after denaturation to produce MS-measurable peptides including possible peptide segments with conformational changes; (**B**) Schematic diagram of the difference in PK hydrolysis after butyric acid bound TGR5. The combination prevented the hydrolysis of PK. (**C**) The volcanic plot of measurable peptide segments in LiP-MS experiments. Significance cutoffs were fold change (FC) > 2 & < 0.5, and p-adjusted (p adj) < 0.05. (**D**) Detailed information on differential peptide segments. LiP-MS, limited proteolysis mass spectrometry; GPBAR1, G protein-coupled bile acid receptor 1; TGR5, Takeda G protein-coupled receptor 5; PK, Protease K; BA, butyric acid

The LiP-MS was performed to explore the direct binding site between butyric acid and TGR5. In this workflow (Fig. 2A-B), TGR5 was incubated with butyric acid or equal volume solvent at 25℃. Limited proteolysis with proteinase K generated structurally specific protein fragments and trypsin was used to digest the fragments, generating peptide mixtures, then samples were injected into LC-MS/MS for further analysis. The sequences of all analyzed peptides were represented through a volcano plot (Fig. 2C), including 54 peptides and the criteria for difference between groups are fold change (FC) > 2 & < 0.5, and p-adjusted (p adj) < 0.05 (Benjamini & Hochberg method). There existed 6 peptides that exhibited statistical differences including GPBAR1 [277–286] [M4], GPBAR1 [275–286], GPBAR1 [315–330], GPBAR1 [277–286], GPBAR1 [275–286] [M6], GPBAR1 [321–330]. GPBAR1 [277–286] [M4] referred to the oxidation modification of the fourth position of methionine in the peptides. GPBAR1 [275–286] [M6] referred to the oxidation modification of the sixth position of methionine in the peptides. The detailed information and statistical results of 6 peptides were presented in the Fig. 2D. From the mass spectrometry data, it can be seen that AVPVAMGLGDQR and IAYHPSSQSSVDDDLN were the main differential peptides, located in the amino acid regions 275–286 and 321–330 of the target protein, respectively, which were potential binding regions for butyric acid.

### Computed analysis of binding modes between TGR5 and butyric acid

**Fig. 3:**
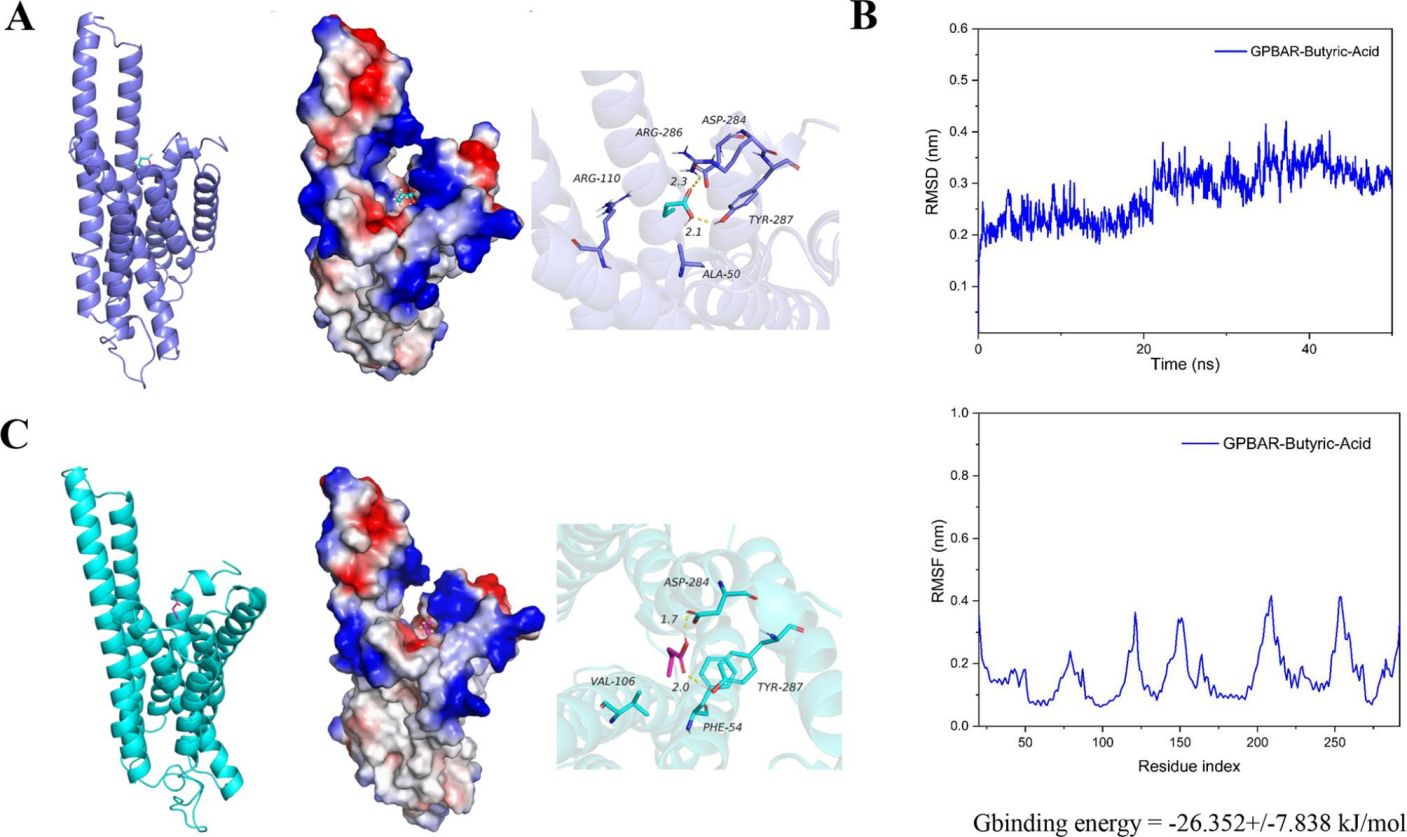
Computed analysis of binding modes between TGR5 and butyric acid. (**A**) The result of molecular docking. (**B**) The RMSD and RMSF of butyric acid with TGR5 and analysis of binding energy. (**C**) Comprehensive analysis of interaction mode. RMSD, Root Mean Square Deviation; RMSF, Root Mean Square Fluctuation; GPBAR1, G protein-coupled bile acid receptor 1; TGR5, Takeda G protein-coupled receptor 5

Based on the results of LiP-MS, we found that regions 275–286 and 321–330 of TGR5 were binding sites for butyric acid, but region 321–330 was located at the end of TGR5. Therefore, region 275–286 was the most likely binding site for butyric acid on the cell. We evaluated the interaction between butyric acid and region 275–286 through molecular docking (Fig. 3A). Butyric acid could form strong hydrogen-bonding interactions with ARG-286 and TYR-287 at the active site of TGR5, with hydrogen-bonding distances of 2.3 Å and 2.1 Å, respectively, which made significant contributions to anchoring butyric acid in the TGR5 pocket. In addition, the hydrophobic end of butyric acid could have hydrophobic interactions with ARG-110 and ALA-50, which played an important role on stabilizing butyric acid. These interactions could effectively promote the formation of a stable complex between butyric acid and TGR5. In molecular dynamics simulations (Fig. 3B), as observed from the RMSD (Root Mean Square Deviation) curve, the displacements of both the TGR5 and butyric acid were small, with both tending to stabilize around 25 ns and converging at the end of the simulation. According to the RMSF (Root Mean Square Fluctuation) plot, a small number of amino acids in the complex formed by the interaction between TGR5 and butyric acid exhibited significant conformational changes. This was primarily attributed to the fact that the protein was primarily composed of several α-helices connected by loops, which were inherently flexible and prone to undergoing certain conformational changes during the simulation. In contrast, the conformations of most amino acids changed less significantly, reflecting the stability of the complex. Gbinding energy was − 26.352+/-7.838 kJ/mol, which highlighted the stability of the system. Based on the above data analysis, butyric acid formed effective hydrogen-bonding interactions with ASP-284 and TYR-287 of TGR5, with hydrogen-bonding distances of 1.7 Å and 2.0 Å, respectively (Fig. 3C).

### SB promoted TGR5/β-arrestin2 expression after LPS administration in RAW264.7 cells

**Fig. 4:**
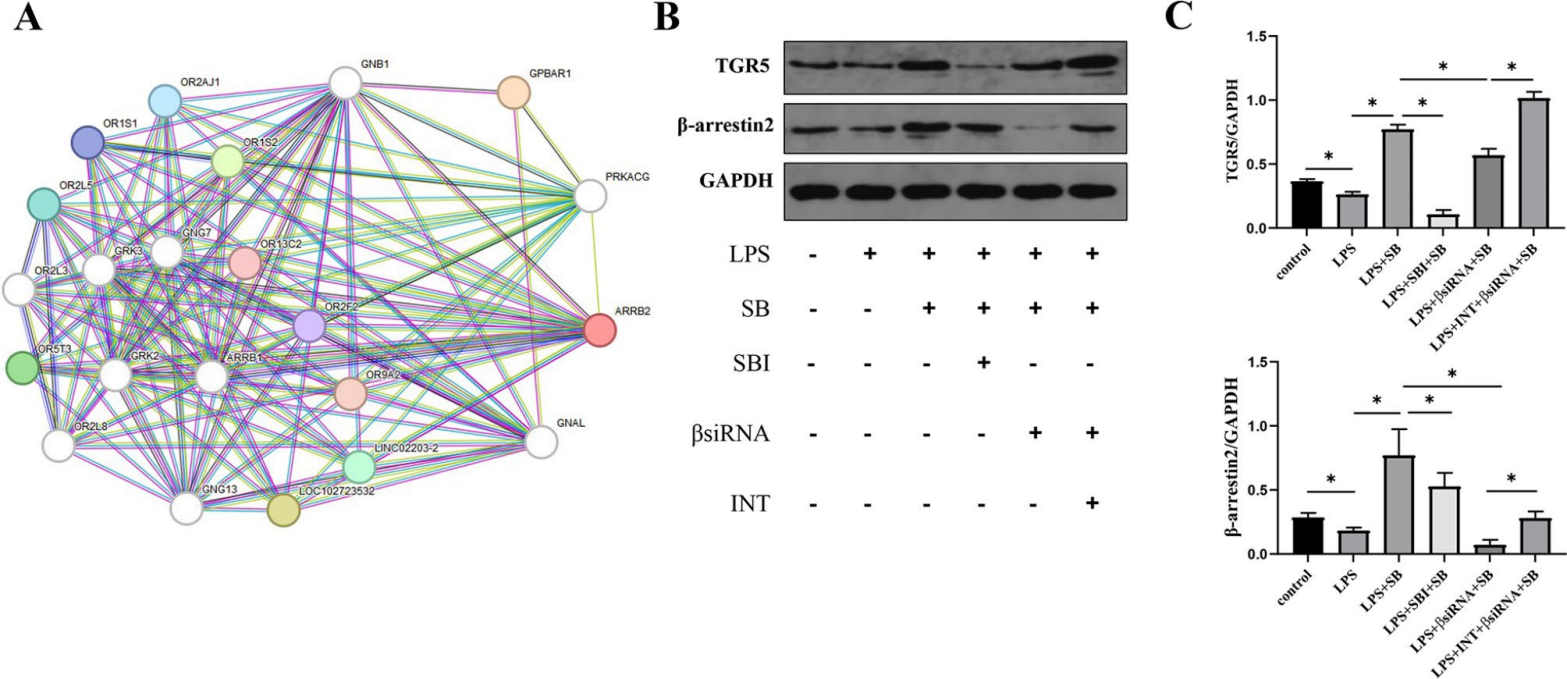
The effect of butyric acid on the TGR5/β-arrestin2 pathway after LPS administration in RAW264.7 cells. (**A**) The relationship between TGR5 and β-arrestin2 in the String database. (**B-C**) Expression levels of TGR5 and β-arrestin2 were detected by western blot. Data were expressed as mean ± standard deviation, *, *P* < 0.05. SB, sodium butyrate; SBI, SBI-115; βsiRNA, siRNA-β-arrestin2; INT, INT-777, TGR5, Takeda G protein-coupled receptor 5

In GPCR signaling, the binding of agonists caused changes in receptor conformation, which in turn leads to the coupling and activation of heterotrimeric G proteins, resulting in the production of second messengers, such as cAMP. TGR5 as a G protein-coupled receptor, had been experimentally determined to interact with G proteins (GNB1) in the STRING database. Although TGR5 might also activate downstream pathways through β-arrestin2, the STRING database did not indicate an interaction between TGR5 and β-arrestin2 (Fig. 4A). To observe the effect of SB on TGR5/β-arrestin2 pathway by western blot, we overexpressed or inhibited TGR5 by adding INT-777 or SBI-115 and knocked down β-arrestin2 by siRNA-β-arrestin2. Compared with the control group, LPS significantly inhibited the expression of TGR5 and β-arrestin2 (*P* < 0.05). SB promoted the protein expression of TGR5 and β-arrestin2 after LPS administration (*P* < 0.05). SBI-115 inhibited the effect of SB on expression of TGR5 and β-arrestin2 induced by LPS (*P* < 0.05). Knockdown of β-arrestin2 also restrained the effect of SB on the expression of TGR5 and β-arrestin2 induced by LPS (*P* < 0.05), but inhibition of β-arrestin2 had a more prominent effect. When we added INT-777, the protein expression of TGR5 and β-arrestin2 increased significantly compared with the simple knockdown of β-arrestin2 (*P* < 0.05). (Fig. 4B-C)

### SB regulated macrophage polarization by TGR5/β-arrestin2 after LPS administration

**Fig. 5:**
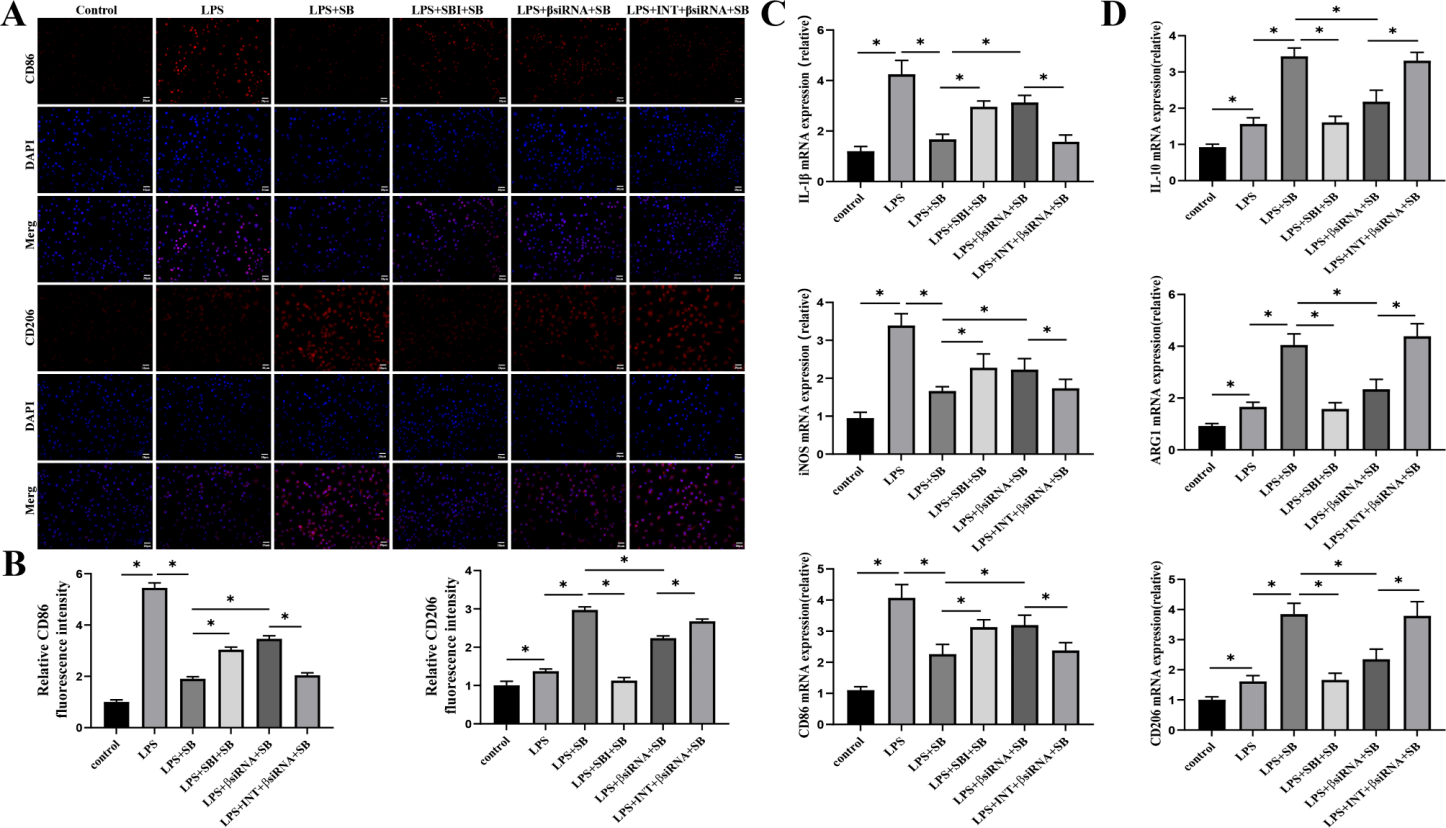
The effect of sodium butyrate on macrophage polarization by TGR5/β-arrestin2 after LPS administration. (**A-B**) Representative florescence pictures of CD86 and CD206 expression in RAW264.7 cells. (**C-D**) qRT-PCR was used to detect expression levels of IL-1β, iNOS, CD86, IL-10, ARG1, and CD206. Data were expressed as mean ± standard deviation, *, *P* < 0.05. SB, sodium butyrate; SBI, SBI-115; βsiRNA, siRNA-β-arrestin2; INT, INT-777, TGR5, Takeda G protein-coupled receptor 5; LPS, lipopolysaccharide; qRT-PCR, Quantitative Real-time Polymerase Chain Reaction

We investigated the inhibition of SB on macrophage polarization through TGR5/β-arrestin2 by immunofluorescence and qRT-PCR. In comparison with the control group, the fluorescence intensity of CD86 and CD206 had increased in LPS group; SB decreased the fluorescence intensity of CD86 and promoted the fluorescence intensity of CD206 induced by LPS. When we added the SBI-115 or used RAW264.7 cells transfected with siRNA-β-arrestin2, the fluorescence intensity of CD86 was increasing and the fluorescence intensity of CD206 was reduced compared with the LPS + SB group. Additionally, INT-777 decreased the fluorescence intensity of CD86 and increased the fluorescence intensity of CD206 in RAW264.7 cells transfected with siRNA-β-arrestin2 after LPS + SB administration. (Fig. 5A-B). Meanwhile, we monitored the mRNA expression of macrophage polarization markers. Then we found that mRNA expression trend of IL-1β, iNOS, and CD86 was consistent with the fluorescence expression of CD86 (Fig. 5C), and mRNA expression trend of IL-10, ARG1, CD206 was consistent with the fluorescence expression of CD206 (Fig. 5D).

## Discussion

UC is a chronic non-specific intestinal inflammatory disease. The incidence of UC is increasing year by year in the world (Du and Ha 2020), and it is prone to recurrence and difficult to cure, which brings heavy burden to individuals and countries. Immune dysfunction is an important pathogenesis of UC (Kondo et al. 2021). Intestinal macrophages are derived from hematopoietic cell and embryonic precursor cell. Blood derived monocyte constantly supplement most of the intestinal macrophages through a series of intermediates. Macrophages are widely accepted for their unique plasticity, which are able to alter their phenotype based on changes in the microenvironment (Mantovani et al. 2005). The macrophages polarize into M1 and M2 cells in response to the local microenvironment of the intestine (Schepper et al. 2018). M1 cells that are sensitive to endotoxin and can be induced by LPS can secrete a large number of inflammatory cytokines, such as IL-1β, IL-6, IL-23, and TNF- α, which can induce local inflammatory response and tissue damage (Mosser and Edwards 2008). M2 cells that are not sensitive to endotoxin and can be induced by IL-4, mainly secreting IL-10, TGF- β and so on, can downregulate local inflammatory response and promote tissue repair (Gordon and Martinez 2010). Gastrointestinal mucosal immune dysfunction is an important pathogenic factor for UC (Chang 2020), and macrophages play a crucial role in preventing excessive immune response (Mowat 2018). LPS can induce M1 polarization, while it can also inhibit M1 polarization by activating Pyruvate Kinase M2 (PKM2), thereby promoting the expression of M2 macrophage-related markers such as IL-10 (Palsson-McDermott et al. 2015). This could also be one of the mechanisms by which macrophages prevent excessive immune responses, or a feedback mechanism of macrophages. In our study, we also observed that LPS increased the expression of M2 macrophage related markers. Butyric acid, as an important metabolite of Clostridium butyricum, exerts a significant influence on macrophage polarization. SB can promote polarization of M2 macrophages induced by phosphatidylserine-containing liposomes and inhibit polarization of M1 macrophages induced by LPS (Wu et al. 2022). Animal model studies have also found that butyrate exerts anti-inflammatory effects on macrophages, and the anti-inflammatory mechanism is unrelated to G protein-coupled receptors, including Gpr109a, Gpr41, and Gpr43 (Chang et al. 2014). However, our study found that TGR5, as a G protein-coupled receptor, regulates macrophage function in response to SB and exerts its effects through the β-arrestin pathway. A recent study suggests that butyrate can promote M2 macrophage polarization and upregulate the expression of CD206 and Arg1 (Liang et al. 2022), which is consistent with the findings of our study, although the specific mechanism has not been explored in depth. In conclusion, butyrate plays an important role in regulating macrophage polarization.

In this study, for the first time, we predicted that TGR5 was a potential target of butyric acid through the SwissTargetPrediction database and confirmed the interaction between them through SPR technology. SwissTargetPrediction database that is an online web tool and available free of charge since 2014 is used to predict the most likely protein targets for small molecules (Daina et al. 2019). The predictive function of SwissTarget Prediction database has been confirmed in many studies. There were 69 resveratrol drug targets in SwissTargetPrediction databases and the target of action of resveratrol was discovered through molecular docking and cell experiment verification (Chen et al. 2023). Using SwissTargetPrediction and SPR analysis, Heat Shock Protein 90 (HSP90) was predicted and verified as a direct binding target of Timosaponin AIII (Zhou et al. 2023). Butyric acid is a very small molecule, and there are very few methods to find its targets. SPR technology, featuring high sensitivity, real-time detection, no need for labeling, and the ability to conduct quantitative analysis, is highly suitable for studying the interaction between proteins and small molecule ligands (Olaru et al. 2015). Yan et al. also confirmed Jolkinolide B interacted with JAK2 to exert anti-inflammatory effects through molecular docking and SPR (Yan et al. 2024). In this SPR experiment, the binding curve between butyric acid and TGR5 showed a steep upward branch which indicated a fast-binding rate and a steep dissociation branch, indicating fast metabolism. The KD value was 13.2 µM, indicating that the binding affinity between butyric acid and TGR5 was moderately above average. In addition, we have established that SB regulated macrophage polarization via TGR5/β-arrestin2 pathway.

In this study, we applied Lip-MS for the first time to detect that the action regions of butyric acid on TGR5 are GPBAR1 [275–286] and GPBAR1 [321–330]. GPCR consists of a single peptide chain, containing seven transmembrane α-helical domains, which divide the receptor into an extracellular N-terminus, three extracellular loops, three intracellular loops, and an intracellular C-terminus. There are binding sites for downstream signaling molecules such as G proteins, GRKs, arrestins, etc., located on the C-terminus of the receptor and the intracellular loop (Lu and Wu 2016). The binding of extracellular ligands to GPCRs leads to conformational changes, which propagate through the seven-transmembrane (7TM) helix bundle, promoting interaction and activation with intracellular transducers (Wingler and Lefkowitz 2020). The rearrangement of the cytoplasmic regions of helices V, VI, and VII of GPCR receptors plays a critical role in the activation process of GPCR (Lu and Wu 2016). A common feature of the cytoplasmic surface of GPCR structures is the chemical environment surrounding highly conserved NPXXY motif residues. The TM7 cytoplasmic end where this motif is located is involved in key conformational changes related to GPCR activation. The proline in this motif causes distortion of the alpha helix structure, and the tyrosine faces a pocket formed by the arrangement of TM2, TM3, TM6, and TM7. The ordered water molecule network in this region is beneficial for enhancing the helical deformation of TM7 and provides hydrogen bonding partners for polar side chains. GPBAR1 [321–330] is located at the C-terminus of the receptor and mainly participates in activating downstream pathways. Therefore, butyric acid is most likely bound to GPBAR1 [275–286] that is located at TM7 (https://www.uniprot.org). Moreover, we found that butyric acid formed effective hydrogen-bonding interactions with ASP-284 and TYR-287 of TGR5 that were located at the cytoplasmic side of TM7, with hydrogen-bonding distances of 1.7 Å and 2.0 Å respectively through molecular docking. In summary, butyric acid is likely to bind to GPBAR1 [275–286] to induce conformational changes in TGR5 and activate downstream pathways on macrophages.

TGR5 is also known as G protein-coupled bile acid receptor 1, which is associated with macrophage polarization. Lithocholic acid exerts anti-inflammatory effects by inhibiting the polarization of M1 macrophages through activating TGR5 (He et al. 2023). TGR5 protects the liver from IR injury by inhibiting pro-inflammatory immune activation through promoting M2 polarization of macrophages (Zhou et al. 2021). There is inconsistency in the relationship between β-arrestin2 and macrophage polarization. Ginsenoside metabolite compound-K promotes the TLR4-Gαs coupling and inhibits overexpressed β-arrestin2 in macrophages, resulting in a decrease in the ratio of M1 to M2 macrophages and improving the prognosis of collagen-induced arthritis mice (Wang et al. 2019). R-salbutamol may inhibit the inflammatory response of M1 macrophages by enhancing the expression of β-arrestin2 and inhibiting the activation of NF-κB (Beng et al. 2023). There are few studies on the relationship between TGR5 and β-arrestin2. TGR5 inhibits the expression of inflammatory mediators by inhibiting the NF-κB pathway through mediating the interaction between IκBα and β-arrestin2 (Wang et al. 2011). In this study, we found that SB promotes the expression of TGR5 and β-arrestin2, accelerates M2 polarization, and inhibits M1 polarization through the TGR5/β-arrestin2 pathway. Another interesting thing is that the knockdown of β-arrestin2 leads to the decrease of TGR5 expression. After TGR5, as a GPCR, is activated by SB, it triggers a series of signal transduction events. β-arrestin2, on the other hand, can bind to activated GPCRs and mediate receptor desensitization, internalization, translocation, and recycling processes. In the absence of β-arrestin2, the internalization and recycling of TGR5 may be compromised, leading to decreased receptor stability on the cell membrane and subsequently causing a reduction in expression levels (Tang et al. 2022).

This study still has some limitations. We have identified the binding of butyric acid to TGR5, but the exact binding mechanism remains unclear. If conditions allow, we can observe the binding site of butyrate and TGR5 using cryo-electron microscopy. It is also possible to further analyze how TGR5 activates downstream signaling through the β-arrestin pathway using cryo-electron microscopy. We have only observed in vitro that butyrate regulates macrophage function through the TGR5/β-arrestin2. The next step could be to use conditional TGR5 or β-arrestin2 knockout mice to further investigate the regulatory mechanism of butyrate-producing bacteria/ butyrate in UC.

In conclusion, our research demonstrates that SB can regulate macrophage polarization by promoting M2 polarization and inhibiting M1 polarization, with the underlying mechanism involving the activation of the TGR5/β-arrestin2 pathway. TGR5 serves as the target of butyrate, while GPBAR1 [275–286] is the specific binding site for butyrate.

## Data Availability

No datasets were generated or analysed during the current study.

## Abbreviations

UC: Ulcerative colitis
SB: Sodium butyrate
SPR: Surface plasmon resonance
Lip-MS: Limited proteolysis mass spectrometry
LPS: Lipopolysaccharide
SBI: SBI-115
βsiRNA: siRNA-β-arrestin2
INT: INT-777
TGR5: Takeda G protein-coupled receptor 5
GPBAR1: G protein-coupled bile acid receptor 1
Arrb2: β-arrestin2
GCPR: G-protein coupled receptor
DSS: Dextran sulfate sodium
HDACI: Histone deacetylase inhibitor
EDC: 1-Ethyl-3-(3-dimethylaminopropyl) carbodiimide hydrochloride
NHS: N-Hydroxysuccinimide
RU: Resonance unit
MD: Molecular dynamics
FBS: Fetal bovine serum
PBS: Phosphate buffered saline
PMSF: Phenylmethanesulfonyl fluoride
TBST: Tris buffered saline containing 0.1% Tween-20
FC: Fold change
PK: Proteinase K
RMSF: Root Mean Square Fluctuation
RMSD: Root Mean Square Deviation
